# Establishing a Novel Group-based Litigation Peer Support Program to Promote Wellness for Physicians Involved in Medical Malpractice Lawsuits

**DOI:** 10.5811/cpcem.1377

**Published:** 2023-10-27

**Authors:** Marla C. Doehring, Christian C. Strachan, Lindsey Haut, Melanie Heniff, Karen Crevier, Megan Crittendon, Jill Nault Connors, Julie L. Welch

**Affiliations:** * Indiana University School of Medicine, Department of Emergency Medicine, Indianapolis, Indiana; † Indiana University School of Medicine, Department of Surgery, Indianapolis, Indiana

**Keywords:** *medical malpractice*, *peer support*, *medical malpractice stress syndrome*, *lawsuit*, *physician wellness*

## Abstract

**Introduction:**

Being named as a defendant in a malpractice lawsuit is known to be a particularly high-stress and vulnerable time for a physician. Medical malpractice stress syndrome (MMSS) is a consequence of being named as a physician defendant in a malpractice lawsuit. Symptoms include depression, anxiety, and insomnia, which may lead to burnout, loss of confidence in clinical decision-making, substance abuse, strain on personal and professional relationships, and suicidal ideation. Although the legal process requires strict confidentiality regarding the specific details of the legal case, discussing the emotional impact of the case is not prohibited. Given that physicians often do not choose formalized therapy with a licensed professional, there is a recognized need to provide physicians with options to support their wellness during a lawsuit.

**Methods:**

The peer support model is a promising option to address the negative impacts to wellness that physician defendants face during medical malpractice lawsuits. We developed and implemented a peer support program to provide a safe, protected space for discussion of the personal impact of a lawsuit and to normalize this experience among peer physicians.

**Results:**

Physicians were receptive to joining a peer support group to discuss the personal impacts of being named in a medical malpractice lawsuit. Participants in this novel group-based program found it helpful and would unanimously recommend it to others who are being sued.

**Conclusion:**

To our knowledge, this pilot study is the first to implement and assess a facilitated, group-based peer support model for emergency physicians who are named as defendants in malpractice lawsuits. While group discussions demonstrated that symptoms of acute distress and MMSS were prevalent among physicians who were being sued, in this study physicians were receptive to and felt better after peer support sessions. Despite increasing burnout in the specialty of emergency medicine (EM) during the study time frame, burnout did not worsen in participants. Extrapolating from this pilot program, we hypothesize that formal peer support offered by EM groups can be an effective option to normalize the experience of being sued, promote wellness, and benefit physicians who endure the often long and stressful process of a medical malpractice lawsuit.

## INTRODUCTION

Physicians are at higher risk of occupational burnout, depression, and suicidal ideation compared to the general population. In high-risk specialties, up to 99% of physicians will be sued for medical malpractice by age 65.^1^ The additional stress from a lawsuit can increase negative impacts to physician mental health.^2^ ^–^ ^4^

Medical malpractice stress syndrome (MMSS) is a constellation of physical and mental symptoms and sequelae that physicians may experience during a medical malpractice lawsuit.^5^ ^,^ ^6^ Examples include anxiety, depression, insomnia, stress on personal/professional relationships, and practicing defensive medicine. Physicians can be reluctant to seek help to address stress or improve their mental well-being. In a recent survey of emergency physicians, almost half endorsed a reluctance to seek mental health resources despite 87% of respondents reporting increased stress and 72% suffering from increased burnout since the beginning of the COVID-19 pandemic.^7^

Findings from a survey of physicians seeking mental health services found that physician colleagues were the preferred source of support (88%) compared to employee assistance programs (29%) and mental health professionals (48%).^8^ In addition, physicians may be reluctant to engage with others at the outset of a lawsuit due to fear of discoverability and advice from their defense counsel to not discuss the legal case details with others. Study participants were told that they could not discuss any details about their actual cases because any conversations other than those with their lawyers or spouses are potentially discoverable in court. However, they were able to discuss their feelings, coping strategies, and general legal processes.

To our knowledge, there is no published literature regarding the use of group-based peer support for physicians who are named in a medical malpractice lawsuit. This feasibility study describes the development and implementation of a novel litigation peer support program for emergency physicians who are defendants in medical malpractice lawsuits and measures physician receptivity to the program. Further, measures of physical and mental symptoms were piloted on a small scale.

## METHODS

### Development and Implementation of Litigation Peer Support Program

The study population was recruited voluntarily from a department of emergency medicine (EM) comprised of more than 200 physicians and non-physician practitioners (NPP) who staff three academic and seven community emergency departments. After department chair approval, a team of key personnel was assembled to develop the program, including the vice-chair for clinical affairs, who was informed of all active lawsuits involving emergency physicians and NPPs; the vice-chair of faculty development, who had experience with the peer support model; an attorney from our institution’s risk retention group (RRG), and emergency physicians with a breadth of expertise in clinical operations, research, and law. The protocol for the litigation peer support program was reviewed and approved by the institutional review board and the RRG.

Emergency physicians and NPPs who were identified as defendants in active lawsuits were contacted confidentially by the vice-chair for clinical affairs. They were invited and consented to participate in a series of voluntary, virtual, one-hour, peer support sessions. Facilitators for the groups were volunteer emergency physician peers who had previously been sued. Facilitator training was created and delivered based upon American Medical Association guidelines for peer facilitators and resources provided by the National Alliance on Mental Illness.^9^ ^–^ ^12^ Facilitator training included both individual preparations lasting approximately three hours and an online group session lasting 90 minutes. New facilitators “shadowed” for one 60-minute peer support session prior to leading a session.

Although the risk of a mental health emergency during a group session is very low, there is potential to trigger traumatic events. A safety plan was developed in case a mental health emergency was identified during a session. A mental health emergency was defined as active suicidal ideation, homicidal ideation, or acute psychosis.

Based on advice from RRG attorneys, physicians involved in the same lawsuit were separated into different cohorts. Although two NPPs were invited to participate in this study, none enrolled. Thus, our study only included physician participants. The program began with two cohorts of 8–10 physicians and two co-facilitators who met monthly during a three-month pilot study.

### Litigation Peer Support Session Structure

The structure of each session was adapted from a model that used group-based peer support for physicians during the COVID-19 pandemic.^11^ Physicians were asked to participate in the sessions from a location that ensured privacy. Facilitators opened with a brief overview of and guidelines for the sessions. Participants agreed to confidentiality and avoidance of any discussion of an active lawsuit. They were reminded that peer support is not formal therapy and that individuals who perceived a need for additional mental health assistance could be referred to appropriate services. Study participants were told that they could not discuss any details about their actual cases because any conversations other than those with their lawyers or spouses are potentially discoverable in court. However, they were able to discuss their feelings, coping strategies, and general legal processes.

**Figure 1.**
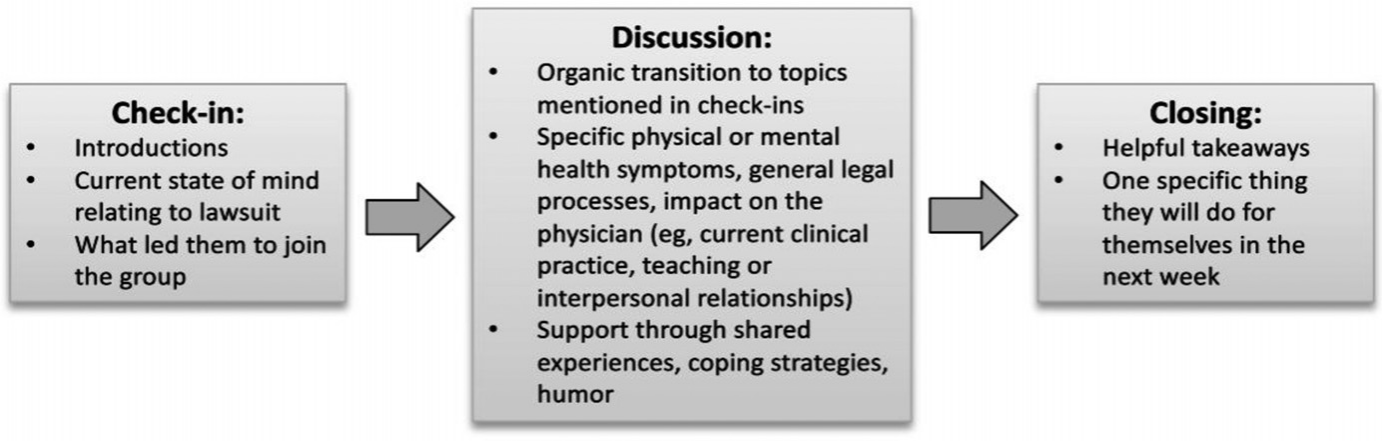
Anatomy of a virtual session for litigation peer support.

The purpose of the sessions was to allow participants a space to process their experiences and use group wisdom to apply principles of support.^11^ To accomplish this, the basic structure of the support group model included three activity components (Figure 1). Each session began with a 2–3 minute “check-in” by each participant. Physicians introduced themselves and briefly summarized their current state of mind related to their lawsuit and/or what led them to join the group. Next, facilitators used supportive communication to transition into an organic discussion that stemmed from topics mentioned by one or more of the participants during check-in. Finally, facilitators transitioned to closing each session positively by inviting each participant to take 1–2 minutes to highlight a helpful takeaway (Figure 1).

### Program Assessment and Data Analysis

To determine the feasibility and receptivity of the program, we used enrollment and attendance rates in addition to a global change rating (GCR) and a net promoter score (NPS) after sessions.^11^ Using customized and validated tools we measured changes in acute distress symptoms and burnout before and after sessions and analyzed the changes using dependent *t*-tests.^13^ ^–^ ^15^ Data was collected via voluntary Qualtrics surveys (Qualtrics International Inc, Provo, UT). A customized checklist tool tracked MMSS symptoms and sequelae discussed during each session.^6^

## RESULTS

**Figure 2.**
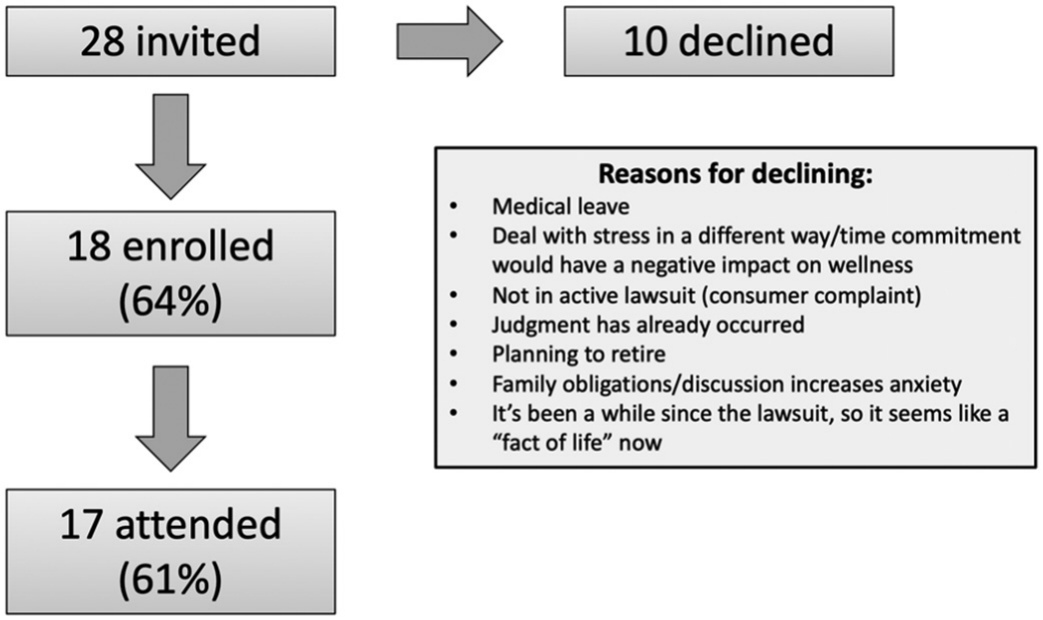
Pilot program feasibility.

A total of 28 physicians and two NPPs with active lawsuits from three academic and seven community EDs were invited to participate in the pilot study. Of the 28 invited, 18 physicians (64%) enrolled, with 17 (61%) attending one to three peer support sessions. Two NPPs and 10 physicians declined participation for variety of reasons (Figure 2). Of the attendees, all were physicians, 41% identified as female, and 24% as non-White.

Receptivity among physicians participating in the program was high. Physicians were willing to discuss 77% of MMSS symptoms and 86% of MMSS sequelae during the sessions. At the end of the sessions, 96% of participants felt better, with the remainder reporting no change on the GCR. At the end of program participation per the NPS, 100% of physician participants would recommend peer support to colleagues being sued. Of the 18 physicians who participated, 15 wanted additional sessions, which subsequently continued quarterly. Several participants expressed interest in becoming facilitators for future groups.

Physician burnout at baseline on the single-item Maslach Burnout Inventory was a mean of 2.93 (scale 0–6), indicating “a few times a month” and remained stable after peer support. At baseline, 73% of physicians reported at least seven of eight acute distress symptoms, with fatigue, anxiety, and insomnia most prevalent. The positive effect size suggested modest improvement in insomnia and depression after peer support but did not reach significance (*P* > 0.1) (Table).

**Table.** Physician burnout and acute distress symptoms pre- and post-peer support sessions (N = 17).

Symptoms	Score range	Mean score pre-intervention (SD)	Mean score post-intervention (SD)	95% CI of the difference	*P*-value^3^	Effect size correlation^4^
Burnout^1^	(0–6)	2.93 (1.62)	3.0 (1.49)	−1.26 to 1.40	0.91	−0.04
Fatigue (tiredness)^2^	(0–10)	4.87 (2.61)	5.30 (2.31)	−1.68 to 2.55	0.68	−0.18
Trouble sleeping (insomnia)^2^	(0–10)	3.0 (2.04)	2.50 (1.84)	−2.16 to 1.16	0.54	**0.26**
Anxiety (nervousness)^2^	(0–10)	3.47 (2.62)	3.30 (1.89)	−2.14 to 1.84	0.88	0.06
Low mood (feeling down)^2^	(0–10)	3.20 (2.31)	3.20 (2.78)	−2.11 to 2.11	1.0	0
Difficulty concentrating^2^	(0–10)	2.87 (2.26)	2.80 (1.87)	−1.86 to 1.72	0.94	0.03
Anger^2^	(0–10)	1.47 (1.46)	1.80 (1.62)	−0.95 to 1.62	0.60	−0.22
Depression (helplessness)^2^	(0–10)	2.60 (2.87)	2.20 (2.66)	−2.76 to 1.96	0.73	**0.15**
Guilt^2^	(0–10)	2.60 (2.44)	3.10 (2.23)	−1.50 to 2.50	0.61	−0.22

1 Single-item Maslach Burnout Inventory.

2 SPADE (sleep, pain, anxiety, depression, and low energy/fatigue) and PROMIS (Patient-Reported Outcomes Measurement Information System) measures.

3 *P*-value <0.05 indicates significance.

4 Positive effect size correlation represents improvement.

*CI*, confidence interval; *SD*, standard deviation.

## DISCUSSION

We were successful in adapting the peer support model, training facilitators, recruiting participants, and providing a safe space to discuss the personal impact of a lawsuit with peers. The groups adhered to the pre-established guidelines during the sessions and no details of active lawsuits were discussed during the sessions. While group discussions demonstrated that symptoms of acute distress and MMSS were prevalent among physicians who were being sued, physicians were receptive to talking about them and felt better after peer support sessions. Despite increasing burnout in the specialty of EM, burnout did not worsen in participants during the study time frame.

This program has the potential of being discoverable during a lawsuit, even if no specific case information is mentioned during a session. Peer support meetings are not legally protected from discovery in the way that privileged conversations such as those between an attorney and client or between spouses are protected. Avoiding mention of case specifics minimizes risk to the defendant. In addition, our attorneys advised that in the unlikely event that a plaintiff attorney inquired about the peer support process, they felt that participation in this program had an extremely unlikely chance of negatively impacting a defendant’s case. In fact, the act of seeking mental health support may positively impact the defendant as it humanizes them, which plaintiff attorneys generally attempt to avoid.

### Lessons Learned

We faced several challenges in implementing this program. More time than anticipated was required to obtain approval. There was some difficulty in gathering an accurate list of defendants named in active lawsuits from the RRG. The logistics of inviting and scheduling participants required significant time from the vice chair for clinical affairs to maintain confidentiality.

Although we did not perform a formal qualitative analysis of the session due to privacy concerns, facilitators did report that younger physicians and those enduring their first lawsuit seemed to be disproportionately impacted when they were named in a lawsuit. Topics of discussion varied between the sessions and included specific physical and mental health symptoms, general legal processes, and the impact of cases on current clinical practice, teaching, or interpersonal relationships. Often the discussions sparked sharing experiences and ideas for coping strategies. Humor was a common addition to the meetings. In the observed group meetings, guilt, anger, frustration, and self-doubt were some of the emotions that were commonly discussed. One participant described their feelings when they learned that a co-defendant had committed suicide. “Suffering in silence,” “gut-punched,” and “shattered confidence” were phrases that physicians used during the sessions to describe their experiences of being sued.

Three of the 10 hospitals represented in this program are academic teaching sites. Anecdotally, we discovered that several physicians from those hospitals who participated in this program felt more comfortable discussing their experiences of being sued with learners. Introducing the uncomfortable topic of the personal impact of being sued may have benefits for medical students, residents, and fellows.

## LIMITATIONS

There are some limitations to this study. Participants included physicians from one organization and two specialties (EM and pediatric EM). While participation was voluntary, it was encouraged by departmental leadership through faculty meetings and newsletters, which may have influenced receptivity. No NPPs chose to participate. While the physicians and NPPs who declined participation provided a variety of reasons, it is possible that some may have been hesitant due to concerns regarding anonymity or discussion of topics that they viewed as stressful or triggering. Although there may be varied opinions regarding future inclusion of NPP defendants who seek support during a medical malpractice lawsuit, our department made a thoughtful decision to offer this program to our NPPs. This decision may be institution-specific and vary depending upon employment models and culture. Finally, the number of participants was relatively small, limiting the power to detect changes in distress or burnout. In addition, the number of sessions offered was limited and may not have been of sufficient strength to anticipate change in these outcomes.

In the future we will offer quarterly sessions, expand the sample size, and track longer term changes in distress, burnout, and wellness factors. In addition, we plan to evaluate barriers to participation, develop train-the-trainer materials, and examine generalizability to other specialties.

## CONCLUSION

Based on this study, formal peer support offered by EM groups is feasible and well received by physicians. Although results of preliminary effectiveness show promise, larger studies need to be conducted to establish that peer support groups can be an effective option to normalize the experience of being sued, promote wellness, and benefit physicians who are defendants in medical malpractice lawsuits.

## References

[1] Jena AB Seabury S Lakdawalla D et al. Malpractice risk according to physician specialty. N Engl J Med. 2011;365(7):629–36. 21848463 10.1056/NEJMsa1012370 PMC3204310

[2] Shanafelt TD Dyrbye LN West CP et al. Suicidal ideation and attitudes regarding help seeking in US physicians relative to the US working population. Mayo Clin Proc. 2021;96(8):2067–80. 34301399 10.1016/j.mayocp.2021.01.033

[3] Balch CM Oreskovich MR Dyrbye LN et al. Personal consequences of malpractice lawsuits on American surgeons. J Am Coll Surg. 2011;213(5):657–67. 21890381 10.1016/j.jamcollsurg.2011.08.005

[4] Kim K Ye GY Haddad AM et al. Thematic analysis and natural language processing of job-related problems prior to physician suicide in 2003–2018. Suicide Life Threat Behav. 2022;52(5):1002–11. 35766392 10.1111/sltb.12896

[5] Sanbar SS Firestone MH.Medical malpractice stress syndrome. In: The Medical Malpractice Survival Handbook. 1^st^ ed. Maryland Heights, MO: Mosby-Elsevier; 2007:9–15.

[6] Reyes C Morrison M Weinstock M. Medical malpractice stress syndrome. Urgent care reviews and perspectives (RAP). 2017. Available at : https://www.hippoed.com/urgentcare/rap/episode/adisposition/medical. Accessed July 2, 2022.

[7] Mental health among emergency physicians. Morning consult. American College of Emergency Physicians. 2020. Available at : https://www.emergencyphysicians.org/globalassets/emphysicians/all-pdfs/acep20_mental-health-poll-analysis.pdf. Accessed September 2, 2022.

[8] Hu YY Fix ML Hevelone ND et al. Physicians’ needs in coping with emotional stressors: the case for peer support. Arch Surg. 2012;147(3):212–7. 22106247 10.1001/archsurg.2011.312 PMC3309062

[9] Creating a peer support program. AMA Steps Forward. 2022. Available at: https://www.ama-assn.org/practice-management/physician-health/creating-peer-support-program. Accessed July 20, 2022.

[10] Peer support resources. National Alliance on Mental Illness (NAMI). 2023. Available at : https://nami.org/Your-Journey/Frontline-Professionals/Health-Care-Professionals/Peer-Support-Resources. Accessed March 7, 2023.

[11] Nault Connors J Thornsberry T Hayden J et al. Brief report: the use of peer support groups for emergency medicine physicians during the COVID-19 pandemic. JACEP Open. 2023;4(1):e12897. 36814587 10.1002/emp2.12897 PMC9939737

[12] *NAMI Family and Friends Leader Manual*. National Alliance on Mental Illness (NAMI). 2017. Available at : https://www.nami.org/NAMI/media/NAMI-Media/downloads/F-F-Leader-Manual.pdf. Accessed September 2, 2022.

[13] Guy W.*ECDEU Assessment Manual for Psychopharmacology*. U.S. Department of Health, Education, and Welfare, Public Health Serivce, Alchohol, Drug Abuse, and Mental Health Administration, National Institute of Mental Health; 1976. Available at: https://ia600306.us.archive.org/35/items/ecdeuassessmentm1933guyw/ecdeuassessmentm1933guyw.pdf. Accessed July 2, 2022.

[14] Kroenke K Stump TE Kean J et al. PROMIS 4-item measures and numeric rating scales efficiently assess SPADE symptoms compared with legacy measures. J Clin Epidemiol. 2019;115:116–24. 31330252 10.1016/j.jclinepi.2019.06.018

[15] West CP Dyrbye LN Sloan JA et al. Single item measures of emotional exhaustion and depersonalization are useful for assessing burnout in medical professionals. J Gen Intern Med. 2009;24(12):1318–21. 19802645 10.1007/s11606-009-1129-z PMC2787943

